# Isolation and selection of cellulose-chitosan degrading bacteria to speed up the mineralization of bio-based mulch films

**DOI:** 10.3389/fmicb.2025.1597786

**Published:** 2025-07-09

**Authors:** Rossana Sidari, Marco Pittarello, Maria Teresa Rodinò, Maria Rosaria Panuccio, Gabriella Lo Verde, Vito Armando Laudicina, Antonio Gelsomino

**Affiliations:** ^1^Department of Agraria, Mediterranea University of Reggio Calabria, Reggio Calabria, Italy; ^2^Department of Agricultural, Food and Forestry Science, University of Palermo, Palermo, Italy

**Keywords:** compost, soil, digestate, earthworms, bacteria, biodegradation, polysaccharide-based film, N and P

## Abstract

**Introduction:**

Mulching is a common agricultural practice owing to its advantages during cultivation. To reduce plastic residues in soil, the use of biodegradable films instead of plastic is desirable. Once buried in soil, biodegradable films undergo degradation driven by abiotic and biotic factors such as the activity of soil microbes. The aim of this study was to select microbial strains whose enzymatic activities can accelerate the degradation of innovative, biodegradable, cellulose-chitosan-based films.

**Methods:**

To this end, 119 bacteria were isolated from compost, digestate, agricultural soil, and the gut or casts of earthworms and subsequently tested for their ability to break down two types of biodegradable films, which were enriched with nitrogen and phosphorus (+NP) and the unenriched control (-NP).

**Results and discussion:**

The ability of the bacteria to accelerate the degradation of the films was strain dependent, and the degradation proceeded at different speeds and degrees. Of the 119 isolated bacteria, 46 strains were able to degrade the unenriched (-NP) film to a greater or lesser extent, with 20 of them able to break it down completely. With regard to the enriched (+NP) film, 10 strains were able to degrade it, with six strains being able to break it down completely. These figures include eight strains that were able to degrade both the enriched and unenriched films. Four novel cellulose-chitosan-degrading bacteria were selected and identified as *Bacillus subtilis* ACT-8, *Bacillus* spp. DL-A1-11, *Pseudomonas* spp. I1, and *Staphylococcus warneri* F7. These strains will be further studied to assess their activity in a mesocosm-scale trial. The novelty of this study is the identification of bacterial strains capable of degrading cellulose-chitosan-based films. This finding contributes to the common effort to reduce the presence of axenic residues in the environment and may have a positive impact on the sector, considering the possibility of applying these strains in bioaugmentation.

## 1 Introduction

Plastic mulch films are among the so-called plasticulture products widely used in agriculture because of their undoubted advantages, such as reduction of water and nutrient loss, effective weed control, increase in crop yield and quality, and reduction of harvest time (Narayan, 2017; Kader et al., 2017; Martín-Closas et al., 2017).

Plastics are not completely biodegradable, and in a short period of time, the process depends on different biotic and abiotic factors (Pauli et al., 2017). Its improper disposal (estimated values of plastic disposed of in landfills or incinerated are 0.67 and 0.37 metric tons/year in Europe, respectively) causes serious environmental pollution (DEGRICOL Project, 2016; He et al., 2015; Teuten et al., 2009; Blettler and Mitchell, 2021). The use of plastic film has increased annually (Reynolds, 2009; Plastics Europe, 2021); however, the aim of the European Union is to reduce waste and create a circular economy (European Commission, 2014).

There is an ever-growing interest in the development of biodegradable polymers that can degrade naturally in the environment. The use of these polymers should be economically sustainable and help solve the problem of pollution. The idea of biodegradable mulch film is not recent; however, its development and large-scale use are linked to its technological and eco-friendly characteristics, which make it suitable both to replace plastic films and allow rapid and complete degradation after landfill disposal at the end of the crop cycle. Indeed, biodegradable plastic mulch films are ideal and promising alternatives to conventional plastics. However, it is still characterized by slow degradation with possible release into the soil and the persistence of micro- and nano-bioplastics and additives (Romano et al., 2024; Campanale et al., 2024).

The duration of the degradation process depends on the climate, polymer features, and soil characteristics, including microbial communities (Kijchavengkul et al., 2008). The latter are affected in their structure and functioning not only by the film mulching action as a surface barrier but also by their chemical composition when buried in the soil (Li et al., 2014; Muroi et al., 2016; Bandopadhyay et al., 2020a; Zhou et al., 2021; Romano et al., 2024; Bernetti et al., 2025). The results of changes in microbial communities are often controversial because of the existence and influence of different conditions, as reported above. Indeed, both an increase and a decrease in microbial activity in the presence of biodegradable plastic films have been reported (Moreno and Moreno, 2008; Sintim et al., 2020; Li et al., 2022). Biodegradable plastic films seem to decrease the number of bacterial species involved in degradation, with a strong depletion of pathogen species compared to polyethylene (Yu et al., 2025) and increased fungal taxa (Bandopadhyay et al., 2018). On the other hand, biodegradable plastic polybutylene adipate-co-terephthalate (PBAT) affects microbial and fungal communities, releasing microparticles through C cycle perturbation (Wang et al., 2025). Interestingly, mulching depletes C mineralization by bacteria in comparison with bare soil (Yang et al., 2025).

To overcome these problems, researchers have considered the use of naturally available polysaccharides such as cellulose, starch, chitin, and alginate (Aleksànyan et al., 2013; Reddy et al., 2013; Menossi et al., 2021; Marasović et al., 2025); microbial biomass and its derivatives (Cottet et al., 2020); novel formulations (Ciaramitaro et al., 2024; Santiago et al., 2024); and novel technologies such as sprayable and biodegradable films (BIOCOAGRI Project, 2015; Santagata et al., 2017). When buried in soil, these novel bio-based mulch films should be degraded completely and rapidly by soil microbial communities, eliminating the release and persistence of plastic residues.

Focusing on the microbiological aspect related to bio-based, in particular cellulose-based, mulch film, there is little knowledge regarding the selection of bacteria for use in rapid degradation. Indeed, recent studies have reported the degradation of cellulose film buried in the soil, observing fungal growth (Rumi et al., 2024), and the degradation of cellulose and cellulose nanocrystals in a mixture with chitosan film by inoculated fungal spores (Escamilla-García et al., 2022).

An interesting approach to accelerate the degradation of bio-based mulch films involves the adoption of a microbial-based technology, which relies on the introduction of single strains or consortia of properly selected highly competitive microorganisms (Mrozik and Piotrowska-Seget, 2010). The novelty of this study is the selection of bacterial strains whose enzymatic properties can be applied to degrade polysaccharide-based materials. In particular, the idea consists of inoculating selected bacteria or a bacterial consortium on the mulch film before it is incorporated into the soil with the aim of accelerating the degradation and mineralization processes. Indeed, bacteria can degrade synthetic and natural polymers because of their enzymatic activities (Pathak, 2017; Urbanek and Rymowicz, 2018). *Bacillus*, *Pseudomonas,* and *Cellulomonas* were reported as cellulose and chitosan degraders (Kameshwar and Qin, 2016; Manzum and Mamun, 2018; Ma et al., 2020), and *Bacillus*, *Pseudomonas*, *Serratia*, *Vibrio*, and *Beauveria* were reported as chitin and chitosan degraders (Makarios-Laham and Lee, 1995; Sawaguchi et al., 2015).

The aim of this study, therefore, is to select bacteria with high cellulolytic and chitinolytic activities for further application in innovative mulch films made of cellulose and chitosan, either enriched or unenriched with an inorganic N- and P-source, thus accelerating their degradation. The selected strains were tested directly on an innovative polysaccharide-based mulch film rather than on a pure polymeric structure. The aim of reproducing the most realistic conditions of the film is to determine strains that can be encountered in the soil and positively condition the microbial community, both in terms of complexity and C mineralization efficiency.

## 2 Materials and methods

### 2.1 Strain isolation

Bacteria were isolated from different environmental sources: (i) composting biomass from municipal solid wastes sampled both in the thermophilic (indicated as ACT) and curing phases (indicated as MAT), (ii) liquid (indicated as DL-A1), and (iii) solid (indicated as DS-A1) fractions of anaerobic digestate after the digestion process of municipal solid wastes containing plastic debris, (iv) an organically managed agricultural soil (indicated as MT) sampled from the Ap layer (5–15 cm), (v) gut (indicated as I), and (vi) casts (indicated as F) of commercial earthworms (*Eisenia fetida* L.) purchased from Biotica (CT, Italy) (Table 1).

**Table 1:** Environmental sources of bacterial isolates tested for their cellulose-chitosan degrading ability.

Sample	Source
Biomasses from the composting process	The composting biomasses (taken at the thermophilic and the curing phases) were kindly provided by a local plant processing the organic fraction of municipal solid waste (MSW) (Calabra Maceri and Servizi S.p.a., Rende, Italy).
Biomasses from the anaerobic digestion process	The liquid and solid fractions of anaerobic digestate were collected at the end of the digestion process (under mesophilic conditions) of the organic fraction of MSW with fragments of MaterBi® (Novamont, Italy) compostable bags.
Agricultural soil	Arable silt loam soil, with a neutral pH and low carbonate content, organically managed under pomegranate cultivation.
Earthworm casts and guts	Commercial individuals of *Eisenia fetida* L. were purchased from Biotica (CT, Italy).

Earthworm guts were obtained following the procedure reported by Liu et al. (2018). Briefly, earthworms were rinsed with tap water, anesthetized with a 70% alcohol solution, washed with sterile deionized water, and dissected with sterile scissors, and the guts were collected.

In order to collect earthworm casts, three or four earthworms were placed on Petri plates containing wet sterile adsorbent paper and left overnight in the dark at room temperature. The casts were collected using a sterile spatula, weighed, and processed as follows:

Solid samples were homogenized in a 1:10 (w/v) sterile 0.9% (w/v) NaCl solution using a Stomacher (Astori) for 2 min at maximum speed. Then, the homogenates and the liquid samples were diluted ten-fold in sterile 0.9% (w/v) NaCl solution and plated by spreading method in triplicate onto Petri plates containing Tryptic Soy Agar (TSA; Sigma Aldrich, St. Louis, USA) and agarized (18 g/L agar) Luria Bertani (LB; Biomaxima, Lublin, Poland) broth for compost, digestate, and agricultural soil; agarized (18 g/L agar) Nutrient Broth (NB; Biomaxima, Lublin, Poland) and Brain Hearth Infusion (BHI; Biomaxima, Lublin, Poland) Agar for earthworm samples. The plates were aerobically incubated at 30°C for 24 h. Subsequently, colonies from each medium were enumerated, randomly chosen, and purified by streaking on the corresponding medium used to isolate them. The plates were aerobically incubated at 30°C for 24 h; then, purified isolates were chosen, grown in broth medium, and stored as glycerol stocks at −80°C until analysis.

### 2.2 Molecular analyses

DNA from the strains was extracted using InstaGene Matrix (Bio-Rad Laboratories) according to the manufacturer’s instructions. Then, 16S rRNA amplification was carried out using fD1 (5′-CCG AAT TCG ACA GAG TTT GAT CCT GGC TCA G-3′) and rD1 (5′-CCC GGG ATC CAA GCT TAA GGA GGT GAT CCA GCC-3′) primers (Thermo Fisher Scientific), according to Martorana et al. (2018).

To avoid testing strain duplicates, the strains were analyzed by Random Amplified Polymorphic DNA (RAPD) using S1508 (AAGAGCCCTC) and S1510 (ACTGCCCGAC) primers (Metabion, Germany), according to Lu et al. (2014). The latter primer was used for strains that did not show a profile with the primer S1508. In detail, the reaction mixture contained 30 ng DNA template, 1 × reaction buffer, 3.0 mM MgCl_2_, 0.2 mM dNTP mix, 0.4 μM of each primer, and 1.0 U of Taq DNA Recombinant (Biotechrabbit). The amplification program was as follows: initial denaturation at 94°C for 3 min; 36 cycles of 30s at 94°C for denaturing, 1 min at 33°C for annealing, 2.5 min at 72°C for extension, and a final extension step of 8 min at 72°C. Amplification was performed using a MasterCycler Nexus GX2 (Eppendorf). After this, agarose (1.5%, w/v) gel electrophoresis amplicons were stained with RealSafe Nucleic Acid Staining Solution (0.5 μL/100 mL) (Real) and checked under UV light (*λ* = 315 nm) (UVITEC Cambridge). The strains with the best performance on the cellulose-chitosan-based film were selected for identification by sequencing the 16S rRNA region (fD1-rD1 primers) according to Weisburg et al. (1991). The amplicons were purified (Illustra GFX PCR DNA and Gel Band Purification Kit, GE Healthcare, UK, Limited) and sequenced by the Sanger method (Eurofins Genomics, Germany). The sequences were analyzed and compared with the sequences of the National Center for Biotechnology Information (NCBI) using BLAST (Altschul et al., 1997) and submitted to GenBank for accession numbers.

### 2.3 Screening of bacterial strains for degradation of cellulose-chitosan-based film

Bacterial strains were tested for their ability to degrade bio-based mulch films made of cellulose and chitosan supplemented with nitrogen and phosphorus in inorganic form (+NP) (90% ammonium phosphate monobasic) or not supplemented (-NP). Briefly, the film was made of 1.5% (w/v) chitosan/sodium alginate and carboxymethylcellulose dispersions at a weight ratio of 1:1. More details about the film and its properties are provided by Ciaramitaro et al. (2024). Subsequently, the strains were grown in the same isolation medium (see Strain Isolation paragraph) overnight at 30°C, centrifuged (5,000 rpm for 10 min) to recover the pelleted biomass, washed once in a sterile 0.9% (w/v) NaCl solution, and resuspended to an OD_600_ of 1.0 in the same solution. Then, each strain suspension was inoculated at 10% in Basal Salt Medium (NaNO_3_ 2.5 g/L, KH_2_PO_4_ 2 g/L, MgSO_4_ 0.2 g/L, NaCl 0.2 g/L, CaCl_2_⋅6H_2_O 0.1 g/L) containing 1 × 1 cm square fragments of the bio-based mulch film made of cellulose and chitosan, both +NP and –NP. Basal Salt Medium with films and without the inoculum was used as a control. The trials were performed in duplicate and incubated at 28°C for 30 days.

During preliminary bacterial inoculation tests, we observed that if the 1 × 1 cm square of film underwent degradation, it started to fragment and became mucilaginous, and therefore difficult to extract from the test tube to be directly analyzed. The degradation of the films by the strains was evaluated by daily macroscopic observations of their integrity, fragmentation, or disappearance.

The best strains were observed under an optical microscope (Olympus BX53, Tokyo, Japan).

### 2.4 Statistical analysis

The bacterial loads (CFU/g) obtained from all the cultivation media were subjected to statistical analysis using Jamovi, version 2.5. As a first step, the dataset was subjected to Shapiro–Wilk and Levene’s tests (*p* < 0.05) to obtain information about dataset normality and homogeneity of variance, respectively. Subsequently, based on these results (normal distribution and non-homogeneity of variance), the dataset was subjected to a one-way Welch ANOVA. The ANOVA yielded significant results; therefore, the Games–Howell *post hoc* test (*p* < 0.05) was applied.

## 3 Results

### 3.1 Bacterial strains and molecular analyses

Table 2 shows the bacterial loads obtained from different matrices. The highest significant load was observed in samples isolated from the compost, in the range of 10^8^–10^9^ CFU/g for both ACT and MAT cultivated in the two media, and in those from earthworm F (10^8^ CFU/g). The lowest load was detected for the digestate, in the range of 10^3^–10^6^ CFU/mL or g for the DL and DS fractions, respectively. Among these two fractions, the DL fraction exhibited a lower load than the DS fraction. Intermediate load values (10^7^ CFU/g) have been reported for agricultural soils and earthworms. The load values were significantly different among all the isolation sources tested in TSA and LB; no significant differences (*p* < 0.05) were observed between the two types of compost used (ACT and MAT), both for the two isolation media used and for the soil tested in both isolation media. Concerning the earthworms, significant differences (*p* < 0.05) were observed among the loads of casts detected in the BHI and loads of guts detected in both NB and BHI. The different isolation media determined load differences (*p* < 0.05) for all the isolation sources, with some exceptions when comparing soil and earthworms.

**Table 2:** Means and standard deviations of microbial loads from the four tested sources.

Media	Sample						
	Compost ACT	Compost MAT	Digestate DL-A1	Digestate DS-A1	Soil MT	Earthworm I	Earthworm F
	(Log CFU/mL)						
TSA	9.75 ± 0.28^a^	9.67 ± 0.42^a^	3.88 ± 0.49^i^	6.28 ± 0.14^f^	7.63 ± 0.57^de^	-	-
LB	8.34 ± 0.07^b^	8.25 ± 0.07^b^	4.00 ± 0.21^h^	5.97 ± 0.07^g^	7.51 ± 1.06^e^	-	-
NB	-	-	-	-	-	7.96 ± 0.78^d^	8.07 ± 0.00^cd^
BHI	-	-	-	-	-	7.93 ± 0.85^d^	8.11 ± 0.14^c^

ACT, compost at the thermophilic phase; MAT, compost at the curing phase; DL, liquid digestate; DS, solid digestate; MT, agricultural soil; I, gut; F, cast; TSA, Tryptic Soy Agar; LB, Luria Bertani; NB, Nutrient Broth; BHI, Brain Heart Infusion. Loads in different media were statistically analyzed together, and different superscript letters indicate significant differences according to the Games–Howell test (*p* < 0.05).

A total of 119 strains were isolated: 55, 30, 16, and 18 from the LB, TSA, NB, and BHI media, respectively. Table 3 shows the sources of isolation of the corresponding bacterial strains obtained.

**Table 3:** Sources of isolation and corresponding bacterial strains.

Source of isolation	Strains
Compost – thermophilic phase	From ACT-1 to ACT-17
Compost – maturation phase	From MAT-18 to MAT-32
Digestate – liquid	From DL-A1-1 to DL-A1-14
Digestate – solid	From DS-A1-1 to DS-A1-12; from DS-A1 14 to DS-A1-20
Agricultural soil	From MT1 to MT17; from MT20 to MT22
Earthworm – gut	From I1 to I9; from I11 to I20
Earthworm – cast	From F1 to F13; from F15 to F18

Concerning the 16S rRNA analysis, for all strains, an expected band of approximately 1,500 bp was obtained, confirming that the strains belonged to bacteria. A total of 95 patterns of RAPD profiles were observed, of which 82 and 13 were obtained using the S1508 and S1510 primers, respectively. These two primers did not yield results for the 17 strains (Table 4). In this analysis, 16 strains showed the same RAPD profile: ACT-10 and MAT-25, ACT-9 and ACT-11, MAT-20 and MAT-27, DL-A1-10 and DL-A1-11, DS-A1-14 and DS-A1-18, MT10 and MT11, I11 and I12, F3, and F11. This profiling allowed for the identification of duplicate strains; therefore, only one of the strains showing the same profile was considered for further analysis (ACT-10, ACT-9, MAT-20, DL-A1-10, DS-A1-14, MT10, I11, and F3). The 17 strains that did not show any profile for either primer were used and further analyzed for their ability to degrade the film.

**Table 4:** All 119 strains: Presence of RAPD profiles using primers S1508 and S1510.

Strain	Primer		Strain	Primer		Strain	Primer		Strain	Primer	
	S1508	S1510		S1508	S1510		S1508	S1510		S1508	S1510
ACT-1	Yes	No	DL-A1-1	Yes	No	MT1	Yes	No	I1	Yes	No
ACT-2	Yes	No	DL-A1-2	Yes	No	MT2	Yes	No	I2	Yes	No
ACT-3	No	Yes	DL-A1-3	No	No	MT3	Yes	No	I3	No	Yes
ACT-4	Yes	No	DL-A1-4	Yes	No	MT4	Yes	No	I4	Yes	No
ACT-5	Yes	No	DL-A1-5	Yes	No	MT5	Yes	No	I5	Yes	No
ACT-6	Yes	No	DL-A1-6	Yes	No	MT6	Yes	No	I7	Yes	No
ACT-7	Yes	No	DL-A1-7	No	No	MT7	No	No	I8	Yes	No
ACT-8	Yes	No	DL-A1-8	Yes	No	MT8	Yes	No	I9	Yes	No
ACT-9	No	*Yes*	DL-A1-9	Yes	No	MT9	Yes	No	I11	No	*Yes*
ACT-10	*Yes*	No	DL-A1-10	*Yes*	No	MT10	*Yes*	No	I12	No	*Yes*
ACT-11	No	*Yes*	DL-A1-11	*Yes*	No	MT11	*Yes*	No	I13	Yes	No
ACT-12	No	Yes	DL-A1-12	No	No	MT12	Yes	No	I14	No	No
ACT-13	No	Yes	DL-A1-13	Yes	No	MT13	Yes	No	I15	No	No
ACT-14	No	Yes	DL-A1-14	Yes	No	MT14	Yes	No	I16	No	No
ACT-15	Yes	No	DS-A1-1	Yes	No	MT15	No	No	I17	Yes	No
ACT-16	Yes	No	DS-A1-2	Yes	No	MT16	No	No	I18	Yes	No
ACT-17	Yes	No	DS-A1-3	No	No	MT17	Yes	No	I19	No	Yes
MAT-18	Yes	No	DS-A1-4	Yes	No	MT20	No	No	I20	Yes	No
MAT-19	Yes	No	DS-A1-5	Yes	No	MT21	No	No	F1	Yes	No
MAT-20	No	**Yes**	DS-A1-6	Yes	No	MT22	No	No	F3	*Yes*	No
MAT-21	No	Yes	DS-A1-7	Yes	No				F4	Yes	No
MAT-22	No	Yes	DS-A1-8	Yes	No				F5	No	No
MAT-23	Yes	No	DS-A1-9	Yes	No				F6	Yes	No
MAT-24	Yes	No	DS-A1-10	Yes	No				F7	Yes	No
MAT-25	*Yes*	No	DS-A1-11	Yes	No				F8	Yes	No
MAT-26	Yes	No	DS-A1-12	Yes	No				F9	Yes	No
MAT-27	No	**Yes**	DS-A1-14	*Yes*	No				F10	No	Yes
MAT-28	No	No	DS-A1-15	Yes	No				F11	*Yes*	No
MAT-29	Yes	No	DS-A1-16	Yes	No				F12	Yes	No
MAT-30	Yes	No	DS-A1-17	Yes	No				F13	Yes	No
MAT-31	No	No	DS-A1-18	*Yes*	No				F15	No	No
MAT-32	Yes	No	DS-A1-19	Yes	No				F16	Yes	No
			DS-A1-20	Yes	No				F17	Yes	No
									F18	Yes	No

The RAPD profiles were considered for groups of strains isolated from the same matrix. In Bold, italics or underlined italics indicate strains with the same molecular profile.

### 3.2 Bacterial ability to degrade the cellulose-chitosan-based film

**Figure 1:**
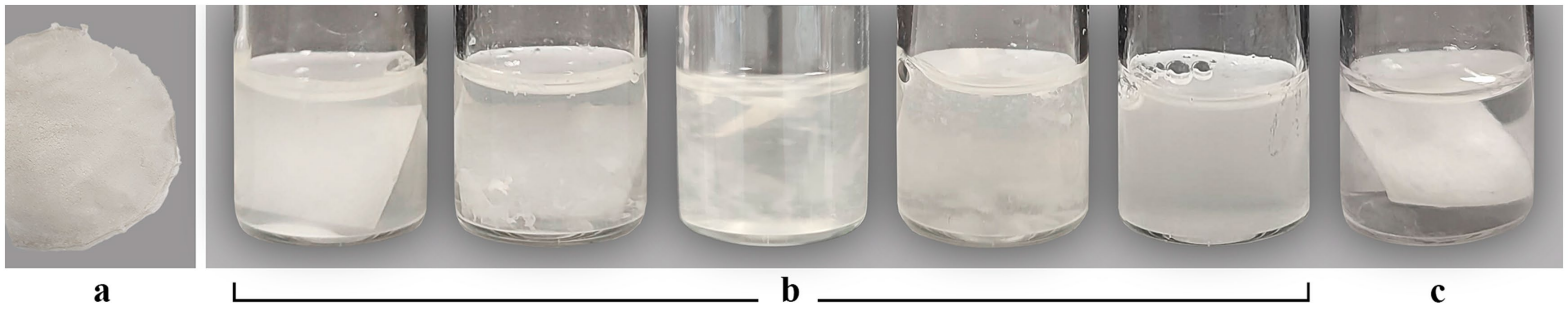
Biodegradability of the cellulose-chitosan-based film **(a)** inoculated with *Bacillus subtilis* ACT-8 **(b)** from 0 to 30 days. Uninoculated samples **(c)**.

Tables 5, 6 report only those strains that showed, over a period of up to 30 days, the ability to degrade cellulose-chitosan-based films to -NP and +NP, respectively. Forty-six strains degraded the -NP film at different speeds and degrees (Table 5). Indeed, the start of degradation was from 4 to 30 days, with a speed from 1 to 16 days and a total time of degradation from 8 to 30 days. Twenty (43%) of these strains completely degraded the film, whereas the remaining 26 strains (57%) were able to partially degrade it. The 1×1 square of the film inoculated with the first group of strains completely disappeared, whereas in tubes containing the film inoculated with the second group of strains, different-sized residues were visible after 30 days of incubation. Figure 1 shows the cellulose-chitosan-based film under study and the biodegradation process over 30 days by the strain ACT-8. In comparison to the control (Figure 1c), it is possible to observe how the entire square of the film progressively fragments until it dissolves completely.

**Table 5:** Strains capable of degrading cellulose-chitosan-based films over 30 days.

Strain	Type of film	First sign of degradation (DAS)^1^	End of observation (DAS)^1^	Fate of the mulch film	Time required for a complete degradation (days)^2^
DL-A1-10	-NP	4	30	Residues	/
ACT-6	-NP	5	30	Residues	/
ACT-8	-NP	5	18	Completely degraded	13
DS-A1-14	-NP	6	30	Residues	/
I1	-NP	6	8	Completely degraded	2
F9	-NP	6	8	Completely degraded	2
ACT-4	-NP	7	30	Residues	/
ACT-15	-NP	7	12	Completely degraded	5
DL-A1-9	-NP	7	30	Residues	/
DL-A1-11	-NP	8	10	Completely degraded	2
I8	-NP	8	15	Completely degraded	7
DL-A1-13	-NP	9	10	Completely degraded	1
I3	-NP	9	15	Completely degraded	6
DS-A1-16	-NP	10	14	Completely degraded	4
MT15	-NP	10	30	Residues	/
ACT-12	-NP	12	18	Completely degraded	6
F4	-NP	13	20	Completely degraded	2
DS-A1-9	-NP	14	30	Completely degraded	16
I18	-NP	15	30	Residues	/
ACT-9	-NP	15	30	Residues	/
MAT-20	-NP	15	20	Completely degraded	5
MAT-21	-NP	15	30	Residues	/
MAT-28	-NP	15	22	Completely degraded	7
MT4	-NP	15	22	Completely degraded	7
MT13	-NP	16	21	Completely degraded	5
MT20	-NP	16	21	Completely degraded	5
F5	-NP	17	30	Residues	/
ACT-2	-NP	19	30	Residues	/
ACT-1	-NP	20	30	Residues	/
ACT-5	-NP	20	30	Residues	/
MAT-29	-NP	20	30	Residues	/
MAT-30	-NP	20	29	Completely degraded	9
MAT-32	-NP	20	30	Residues	/
I2	-NP	20	30	Residues	/
I7	-NP	20	30	Residues	/
F10	-NP	20	30	Residues	/
DL-A1-8	-NP	20	30	Residues	/
DS-A1-17	-NP	22	30	Completely degraded	8
I11	-NP	27	30	Residues	/
F16	-NP	27	30	Completely degraded	
I15	-NP	28	30	Residues	/
I20	-NP	28	30	Residues	/
F3	-NP	28	30	Residues	/
DS-A1-1	-NP	28	30	Residues	/
MT16	-NP	28	30	Residues	/
DS-A1-2	-NP	30	30	Residues	/

^1^Days after the start of the trial; ^2^difference between the end of observation and the start of degradation.

**Table 6:** Strains capable of degrading cellulose-chitosan-based films enriched with N and P over 30 days.

Strain	Type of film	First sign of degradation (DAS)^1^	End of observation (DAS)^1^	Fate of the mulch film	Time required for a complete degradation (days)^2^
ACT-3	+NP	12	19	Completely degraded	7
ACT-4	+NP	13	27	Completely degraded	14
ACT-6	+NP	13	19	Completely degraded	6
ACT-8	+NP	13	19	Completely degraded	6
F7	+ NP	13	22	Completely degraded	9
I1	+ NP	15	30	Residues	/
ACT-15	+NP	15	30	Residues	/
F3	+NP	17	19	Completely degraded	2
ACT-5	+NP	20	30	Residues	/
ACT-2	+NP	27	30	Residues	/

^1^Days after the start of the trial; ^2^difference between the end of observation and the start of degradation.

Ten strains degraded the +NP film at different speeds and degrees (Table 6). These strains began to degrade the film more slowly than the -NP film. The start of degradation was from 12 to 27 days, with a speed from 2 to 14 days and a total time of degradation from 19 to 27 days. Six (60%) of these strains completely degraded the film, whereas four (40%) were able to partially degrade the film, leaving visible residues.

**Figure 2:**
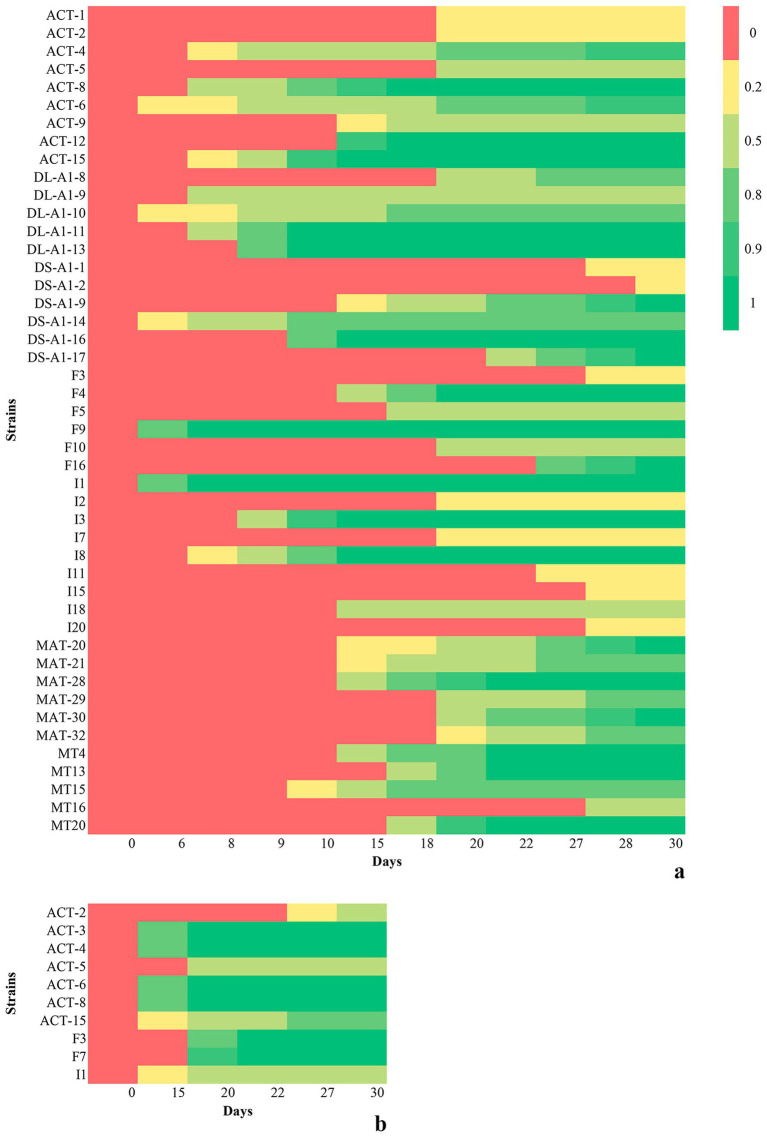
Heatmap summarizing the bacterial degradation of cellulose-chitosan-based film without **(a)** and with **(b)** N and P over 30 days.

Figure 2 shows the progression of cellulose-chitosan-based film degradation by the tested strains over 30 days. The degree of degradation, assessed by macroscopic observation, was assigned as 0 = entire film, 0.2 = large fragments, 0.5 = medium fragments, 0.8 = small fragments, 0.9 = tiny fragments, and 1 = no film residues. The heatmap colors allow the degradation process for each strain to be easily followed; for some of them, it is possible to stop the incubation very early in the experimental period because of the complete degradation of the films (see also Tables 5, 6).

Eight strains—ACT-2, ACT-4, ACT-5, ACT-6, ACT-8, ACT-15, F3, and I1—degraded both -NP and +NP films, with different degrees of degradation for the two types of films (Tables 5, 6).

Taking into consideration both the speed of the strains in attacking the film and their ability to completely degrade it, four strains were selected as the best performing: ACT-8, DL-A1-11, I1, and F7. The strains ACT-8, DL-A1-1, and I1 were rod-shaped and arranged in diplobacilli or chains, whereas F7 was a coccus-shaped bacterium.

### 3.3 Molecular identification of the best strains

Strain ACT-8 was identified as *Bacillus subtilis* (GenBank accession number: PV589511), and strain DL-A1-11 was identified as *Bacillus* spp. (GenBank accession number: PV589512), and strain I1 was identified as *Pseudomonas* spp. (GenBank accession number: PV589513). Considering the percentage identity obtained by BLAST for the latter two strains, further analyses are necessary to assign them to a species. Strain F7 was identified as *Staphylococcus warneri* (GenBank accession number: PV589514).

## 4 Discussion

### 4.1 Degradation ability of the bacteria

The great versatility of microorganisms in decomposing xenobiotics and natural compounds due to their enzymatic activities is well known. Bacteria have been isolated from contaminated industrial sites, plastics buried in soils, coffee exocarps, aquaculture ponds, and earthworms (Villaverde et al., 2019; Bui, 2014; Dat et al., 2019; Fujii et al., 2012) to obtain strains capable of reducing environmental pollution by decomposing plastic, bioplastic, and organic wastes.

In this study, bacteria from different sources (soil, compost, digestate, earthworm guts, and casts) capable of partially or completely degrading cellulose-chitosan-based films were isolated. This ability was strain-dependent; moreover, the +NP film delayed the start of the degradation. This could be because the strains first used the nitrogen and phosphorus released in the solution before attacking the film.

There has been a continuous search for good microbial degraders of different polymers, both synthetic and natural. To the best of our knowledge, no study has reported the selection of bacteria capable of degrading cellulose-chitosan in the film form. The ability of bacteria to degrade cellulose has been tested using filter papers on Petri plates, or the pure polymer carboxymethylcellulose and Red Congo, and the pure polymer chitosan to evaluate its degradation (Gupta et al., 2012; Egwuatu and Appeh, 2018; Behera et al., 2014; Yabuki et al., 1987; Yoshihara et al., 1990; Chasanah et al., 2009). Filter papers were compared to Mater-Bi® EF04P degradation in the soil, and they completely disappeared after 119 and 364 days, respectively (Tosin et al., 2020). Recently, pure microcrystalline has been used as a reference polymer to evaluate the effects of Mater-Bi® EF04P on soil microbial communities (Bernetti et al., 2025).

Hence, the innovation of our study lies in having selected bacterial strains and testing them directly on a mulch film containing both cellulose and chitosan, both +NP and -NP, thereby providing the strains with the most realistic conditions for the film that they may encounter in the soil.

Microorganisms for the degradation of biodegradable plastics, such as poly(butylene succinate) (PBS), poly(butylene succinate)-co-(butylene adipate) (PBSA), poly(L-lactic acid) (PLA), poly(butylene terephthalate) (PBT), PBAT, and polyhydroxyalkanoate (PHA), have been reported and tested on pure polymers, plastic emulsions, or films (Shah et al., 2014; Rüthi et al., 2023). Bandopadhyay et al. (2020b) reported the deterioration of pieces of biodegradable mulch film incubated in soil extracts from which they isolated bacteria, among which *Pseudomonas* sp. A strain of *Pseudomonas putida* isolated from a biodegradable film buried in soil showed the ability to grow in the presence of biodegradable films under laboratory conditions (Fontanazza et al., 2021). PBAT mulch film was degraded at different rates by the S2313 strain of *Peribacillus frigoritolerans* (Wufuer et al., 2022) and the L3 strain of *Pseudomonas sichuanensis* (Chen et al., 2025). Moreover, enzymes purified from *Roseateles depolymerans* strain TB-87 showed activity against poly[(butylene succinate/terephthalate/isophthalate)-co-(lactate)] (PBSTIL) in emulsified and film forms (Shah et al., 2013).

Four novel strains—*Bacillus subtilis* ACT-8, *Bacillus* spp. DLA1-11, *Pseudomonas* spp. I1, and *Staphylococcus warneri* F7—selected here as the best degraders of the cellulose-chitosan-based film, belong to species and genera in agreement with previous research evaluating the enzymatic properties of bacteria. *Bacillus* spp., *Bacillus subtilis*, and *Pseudomonas* spp. have been reported as producers of cellulase and chitosanase and are therefore able to degrade cellulose and chitosan (Ray et al., 2007; Acharya and Chaudhary, 2012; Bui, 2014; Khianngam et al., 2014; Khatiwada et al., 2016; Reddy et al., 2017). The strain *Staphylococcus warneri* F7, isolated from *E. fetida* L., has been reported as an inhabitant of the earthworm gut (Pérez-Pérez et al., 2018) and is able to degrade cellulose (Pourramezan et al., 2012; Manfredi et al., 2015).

The cell morphologies of the four selected strains (see Subsection 3.2) confirmed the species and genera (*Bacillus subtilis*, *Bacillus* spp., *Pseudomonas* spp., and *Staphylococcus warneri*) obtained by sequencing.

Considering the need to avoid soil pollution, the strains selected here could be useful because they, at least *in vitro*, rapidly start (5–13 days) and conclude (2–13 days) the film degradation process. Films made of cellulose, chitosan, and filter paper were tested for degradation after inoculation with different species of fungi. After 10 days and progressively until 28 days, the area of cellulose film and filter papers covered by mycelia increased, whereas no fungal growth was observed on chitosan films (Escamilla-García et al., 2022). In experiments on soil-buried cellulose films, the time reported until complete degradation was 63 days, with the appearance of fungal mycelia after 35 days of burial (Rumi et al., 2024). These results are not comparable to ours because of the different aims, methods, and microorganisms used; however, they indicate an interest in the biodegradation of this type of film.

### 4.2 Practical implications of this study

The challenge of this research was to find a simple and rapid way to test and screen a high number of bacterial strains *in vitro* to select the best strains for cellulose/chitosan-based film hydrolysis. This was paramount because the present research is part of a larger project in which an important goal is the rapid biological degradation of a novel polysaccharide-based mulch film. Four novel strains, *Bacillus subtilis* ACT-8 and *Bacillus* spp. DLA1-11, *Pseudomonas* spp. I1, and *Staphylococcus warneri* F7, are good candidates as a solution to the improper disposal of films used in the field after a crop cycle involving mulching.

Future research will be focused on testing the four selected strains using compatibility tests in Petri plates in order to establish a bacterial consortium. It will be tested in enclosed soil systems, such as mesocosm units, covered with a cellulose-chitosan-based film in order to mimic the agricultural practices used at the end of the mulching period. Subsequently, at the end of this period, the film will be removed, and the microbial consortium will be added before the film is buried. This could speed up film degradation with the benefit of reducing the permanence of mulch film debris in soil.

Furthermore, as the introduction of novel strains into a habitat affects both the phylogenetic composition and activity levels of autochthonous microbial communities (Bandopadhyay et al., 2020a), it is essential to verify the effectiveness of the newly introduced consortium and its ability to compete with the native soil microbial community.

## 5 Conclusion

In the present study, 119 strains of bacteria were isolated and screened for their ability to degrade cellulose-chitosan-based films, both with and without supplementation with nitrogen and phosphorus (+NP and -NP). Eight of the 119 strains degraded both the +NP and -NP films. Four of the 119 strains—identified as *Bacillus subtilis* ACT-8, *Bacillus* spp. DLA1-11, *Pseudomonas* spp. I1, and *Staphylococcus warneri* F7—were selected as the most suitable for degrading the films. *In vitro,* these strains were able to rapidly start (from 5 to 13 days) and complete (from 8 to 22 days) the degradation process of the cellulose-chitosan-based film, i.e., without leaving film residues in the experimental medium. These characteristics of the strains are reflected in the speed of the degradation process (from 2 to 9 days); therefore, these strains may also rapidly degrade the film in the environment. Moreover, we demonstrated that N and P supplementation of the film affected the strain degradation. This finding may be useful when choosing mulch components to avoid inhibiting the hydrolytic activity of microorganisms present in the soil. Considering the need for a reduction in plastic/bio-based plastics in the soil, circular economy, and sustainable agriculture, these strains could be useful individually or as a consortium to accelerate the mineralization of polysaccharide-based mulch films.

## Data Availability

The data presented in this study are deposited in GenBank repository (https://www.ncbi.nlm.nih.gov/genbank/), accession numbers PV589511, PV589512, PV589513, PV589514.
